# BcEaf2 plays a critical role in hyphal growth, asexual reproduction, pathogenic infection, and histone methylation in *Botrytis cinerea*

**DOI:** 10.3389/fmicb.2026.1978891

**Published:** 2026-10-20

**Authors:** Junwei Wu, Weigang Wu, Yajing Wu, Jianyu Hao, Wenchao Chen, Haixu Wang, Chen Yang, Fengying Shen

**Affiliations:** 1 College of Agriculture and Forestry Science and Technology, Hebei North University, Zhangjiakou, China; 2 Hebei Academy of Forestry and Grassland Science, Shijiazhuang, China; 3 Zhangjiakou Academy of Agricultural Sciences, Zhangjiakou, China

**Keywords:** *Botrytis cinerea*, H3K36me3, SANT domain, stress tolerance, virulence

## Abstract

Histone modifications are central to the regulation of fungal development and pathogenicity, yet the upstream mechanisms modulating these epigenetic marks remain incompletely understood in phytopathogenic fungi. Here, we identify and functionally characterize BcEaf2, a conserved Eaf2/DMAP1 homolog, in the necrotrophic pathogen *Botrytis cinerea*. *BcEaf2* gene exhibits dynamic expression during conidiation and host infection, and its silencing results in impaired hyphal growth, heightened sensitivity to oxidative and osmotic stresses, reduced conidiation, and markedly attenuated virulence. Notably, silencing of *BcEaf2* leads to a specific decrease in global H3K36me3 levels, without affecting other major histone modifications, indicating a selective role in chromatin regulation. Mechanistically, we demonstrate that BcEaf2 physically interacts with the H3K36 methyltransferase BcSET2 and significantly enhances its enzymatic activity *in vitro*. Consistent with this, deletion of *BcSET2* phenocopies *BcEaf2*-silenced strains, displaying defects in growth, stress adaptation, conidiation, and pathogenicity, alongside reduced H3K36me3 levels. These findings establish BcSET2 as a key regulator of fungal fitness and virulence and reveal BcEaf2 as a cofactor that fine-tunes BcSET2-mediated histone methylation. Collectively, our study uncovers a previously unrecognized regulatory module controlling H3K36me3 deposition in *B. cinerea* and highlights a functional divergence of Eaf2 proteins from canonical roles in histone acetylation or DNA methylation toward direct modulation of histone methyltransferase activity. These findings reveal a critical role of the BcEaf2/BcSET2 regulatory module in fungal development and pathogenicity and provide a mechanistic framework for exploring this module as a potential target for future antifungal intervention.

## 1 Introduction

*Botrytis cinerea* is a ubiquitous airborne pathogen infecting over 500 plant species across temperate and subtropical regions (Chen T. et al., 2023; Dean et al., 2012; van Kan et al., 2014). Traditionally characterized as a necrotroph, it rapidly penetrates host tissues and promotes cell death through a diverse arsenal of phytotoxic factors–including secondary metabolites, reactive oxygen species (ROS), and necrosis-inducing proteins-as well as cell wall-degrading enzymes; however, emerging evidence indicates that it can also establish asymptomatic endophytic colonization (Cantu et al., 2008; Choquer et al., 2021). During its life cycle, *B. cinerea* produces conidiophores and conidia for dispersal under favorable conditions, or forms melanized sclerotia as survival structures under adverse environments (Espino et al., 2010; Jiao et al., 2022; Prusky and Yakoby, 2003; Weiberg et al., 2013). These sclerotia exhibit developmental plasticity, giving rise to either asexual structures (mycelia and conidia) or, following fertilization, sexual reproductive structures (apothecia and ascospores), although the latter occurs infrequently in natural populations (Cessna et al., 2000). Light serves as a key environmental cue regulating morphogenesis, promoting conidiation while repressing sclerotial formation, though substantial phenotypic variability exists among field isolates (Williams et al., 2011). Owing to its broad host range and economic importance, *B. cinerea* has emerged as a model system for investigating fungal pathogenicity, fungicide resistance, developmental regulation, and plant immune responses (Prusky et al., 2013). The availability of a reference genome, together with efficient homologous recombination and well-established reverse genetic tools, has facilitated functional characterization of numerous genes implicated in host–pathogen interactions, including those involved in toxin production, ROS homeostasis, transport processes, and signal transduction pathways (Billon-Grand et al., 2012).

Epigenetic regulation has emerged as an important mechanism linking chromatin organization to fungal development and pathogenicity. In plant-pathogenic fungi, diverse chromatin-associated processes, including DNA methylation, histone acetylation, histone methylation, chromatin remodeling, and ATP-dependent nucleosome regulation, have been implicated in the control of developmental transitions and host infection (Dubey and Jeon, 2017). For example, histone acetylation and deacetylation can modulate the expression of genes required for conidiation, appressorium formation, secondary metabolism, and host colonization in several fungal pathogens. Histone methylation is also increasingly recognized as an important regulator of fungal pathogenicity. Histone methyltransferases and demethylases targeting residues such as H3K4, H3K9, H3K27, and H3K36 can influence transcriptional programs associated with fungal development, stress adaptation, and virulence (Lai et al., 2022). In *Magnaporthe oryzae*, *Fusarium* species, *Botrytis cinerea*, and other plant-pathogenic fungi, perturbation of chromatin-modifying enzymes has been shown to affect infection-related development and pathogenicity (Kong et al., 2018; Lin et al., 2021; Wang et al., 2020). However, most previous studies have focused on individual histone-modifying enzymes or specific histone marks, whereas how chromatin-associated regulatory factors cooperate with histone methyltransferases to establish transcriptional states that coordinate fungal growth, development, and pathogenicity remains less well understood. In particular, the molecular mechanisms linking H3K36 methylation to pathogenicity in *B. cinerea* and the upstream chromatin-associated factors that regulate this pathway remain poorly characterized.

DNA methyltransferase 1-associated protein 1 (DMAP1) was initially identified as a cofactor of DNMT1, interacting with its N-terminal domain to promote transcriptional repression (Lee et al., 2010; Rountree et al., 2000). The DMAP1-DNMT1 complex further cooperates with the p33ING1-Sin3-histone deacetylase 2 (HDAC2) complex to maintain pericentric heterochromatin integrity during late S phase through coordinated histone deacetylation, histone H3 lysine 9 methylation, and DNA 5mC deposition. These chromatin-modifying activities are essential for the formation of replication foci and proper cell-cycle progression across the G1/S and G2/M transitions (Rountree et al., 2000; Xin et al., 2004). In addition, DMAP1 is a core component of the TIP60-p400 complex, a multifunctional histone acetyltransferase and chromatin remodeling assembly (Cai et al., 2003; Doyon et al., 2004). Notably, DMAP1 also plays a positive role in the DNA damage response by facilitating ataxia telangiectasia mutated (ATM)-mediated repair pathways (Penicud and Behrens, 2014). Yeast Eaf2, a homolog of human DMAP1, functions as a core subunit of the NuA4 histone acetyltransferase complex and contains a conserved SANT domain associated with epigenetic regulation (Ji et al., 2022). However, its role in *B. cinerea* remains largely unexplored. Whether Eaf2 contributes to chromatin dynamics and pathogenicity in filamentous plant pathogens therefore remains an open question. Here, we sought to define the function of Eaf2 in *B. cinerea* and its role in fungal development and virulence.

## 2 Results

### 2.1 BcEaf2 and its gene expression profile in *B. cinerea*

To explore the molecular and biological functions of Eaf2 in *B. cinerea*, we performed a genome-wide search using a hidden Markov model-based approach. A putative Eaf2 homolog was identified and subsequently designated as *BcEaf2* (Supplementary Table 1). Phylogenetic analysis incorporating Eaf2 protein sequences from representative eukaryotic lineages revealed that oomycete orthologs are highly conserved and form a distinct clade, clustering more closely with those from algae and plants than with counterparts from animals and fungi (Figure 1A). *BcEaf2* encodes a 568-amino-acid protein harboring a conserved SANT domain, a hallmark feature retained across orthologs in diverse model organisms (Figure 1B).

**FIGURE 1:**
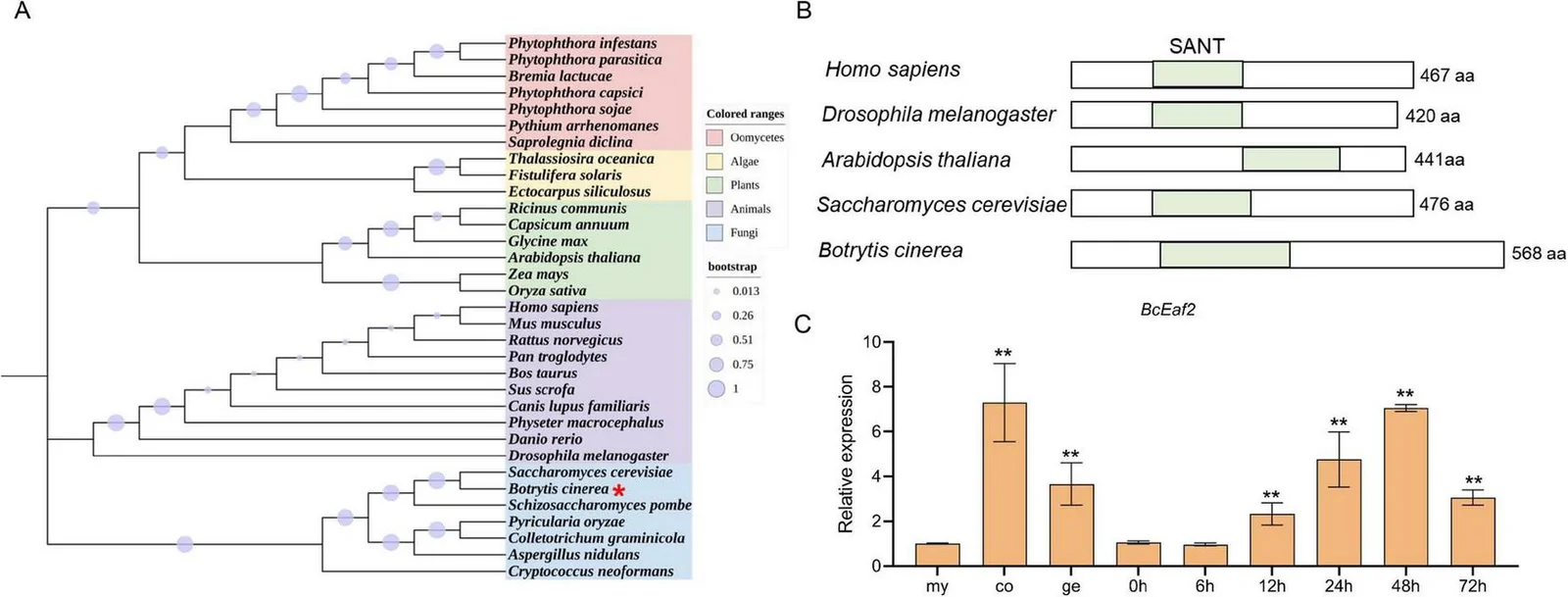
Phylogenetic analyses based on the Eaf2 protein and the transcriptional profile of the *BcEaf2* gene in the life cycle of *Botrytis cinerea*. **(A)** Phylogenetic tree based on Eaf2 sequences from representative species of animal, plant, algae, fungi, and oomycetes. **(B)** Eaf2 domain structures in *Homo sapiens*, *Drosophila melanogaster*, *Arabidopsis thaliana*, *Saccharomyces cerevisiae*, and *B. cinerea*. Eaf2 contains a classical SANT domain. **(C)** Expression patterns of the *BcEaf2* gene at different stages including mycelium (my), conidia (co), germination (ge), and at 0, 6, 12, 24, 48, and 72 h post-inoculation. Data are presented as the mean ± standard deviation from three biological replicates. Statistical significance of above strains compared to the mycelium was determined using unpaired two-tailed *t*-tests (**P < 0.01). The red asterisk (*) indicates the target species, *Botrytis cinerea*.

To gain insight into its potential functional roles, the transcriptional dynamics of BcEaf2 were examined across multiple developmental and infection stages. BcEaf2 displayed stage-specific expression patterns, with transcript levels elevated in conidia and during germination relative to the mycelial stage, which was used as a baseline reference. Notably, BcEaf2 expression was further upregulated during host infection, with peak levels observed at the later stages of invasion (Figure 1C).

### 2.2 Mycelial growth of BcEaf2-silenced transformants under various conditions

Despite the exhaustive screening of over 300 transformants, our attempts to generate homozygous null mutants of *BcEaf2* via homologous recombination remained unsuccessful. Given that loss of murine DMAP1 (the mammalian ortholog of Eaf2) results in embryonic lethality (Boulanger et al., 2024), We therefore hypothesized that complete loss of BcEaf2 may cause a severe defect in the viability or recovery of *B. cinerea* transformants. To circumvent this potential limitation, we generated *BcEaf2*-silenced strains (Supplementary Figures 1A,B), designating three independent mutants (siBcEaf2-1, siBcEaf2-2, and siBcEaf2-3) for downstream functional analysis, alongside EV control WT expression levels. To quantitatively assess the efficiency of *BcEaf2* silencing, the transcript levels of *BcEaf2* were determined in three independent silenced mutants by quantitative RT-qPCR. Compared with the WT strain, *BcEaf2* transcript levels were reduced by approximately 56%, 52%, and 55% in siBcEaf2-1, siBcEaf2-2, and siBcEaf2-3, respectively (Supplementary Figure 1B). These results confirmed comparable and reproducible silencing of *BcEaf2* among the three independent mutants. Because a specific antibody against endogenous BcEaf2 is currently unavailable, the corresponding changes in endogenous BcEaf2 protein abundance could not be directly determined. To verify that the observed phenotypes were specifically caused by *BcEaf2* silencing, *BcEaf2* was reintroduced into the siBcEaf2-1 transformant by overexpression, and the complemented strain siBcEaf2-C was successfully obtained (Supplementary Figure 1B). Although colony morphology remained largely unchanged, all silenced strains displayed a pronounced reduction in radial growth relative to both wild-type and EV controls (Figures 2A,B). To clarify whether *BcEaf2*-silenced strains exhibit delayed growth or growth arrest, we performed additional time-course analyses of mycelial growth by measuring colony diameters at 1, 3, and 6 days post-inoculation. As shown in the revised Figures 2A,B, the *BcEaf2*-silenced strains displayed a consistently and significantly reduced growth rate throughout the observation period. Notably, by 6 days, the wild-type, EV, and complemented strains had completely covered the Petri dishes, whereas the *BcEaf2*-silenced strains still failed to fully occupy the plates, indicating a persistent growth defect rather than a temporary developmental delay (Figures 2A,B). Furthermore, Microscopic observation showed that the *BcEaf2*-silenced strains exhibited a markedly sparser hyphal network than the wild-type, EV, and complemented strains. The hyphae were less densely distributed, with fewer branches and larger gaps between neighboring hyphae, whereas the complemented strain largely restored the wild-type phenotype (Figure 2C).

**FIGURE 2:**
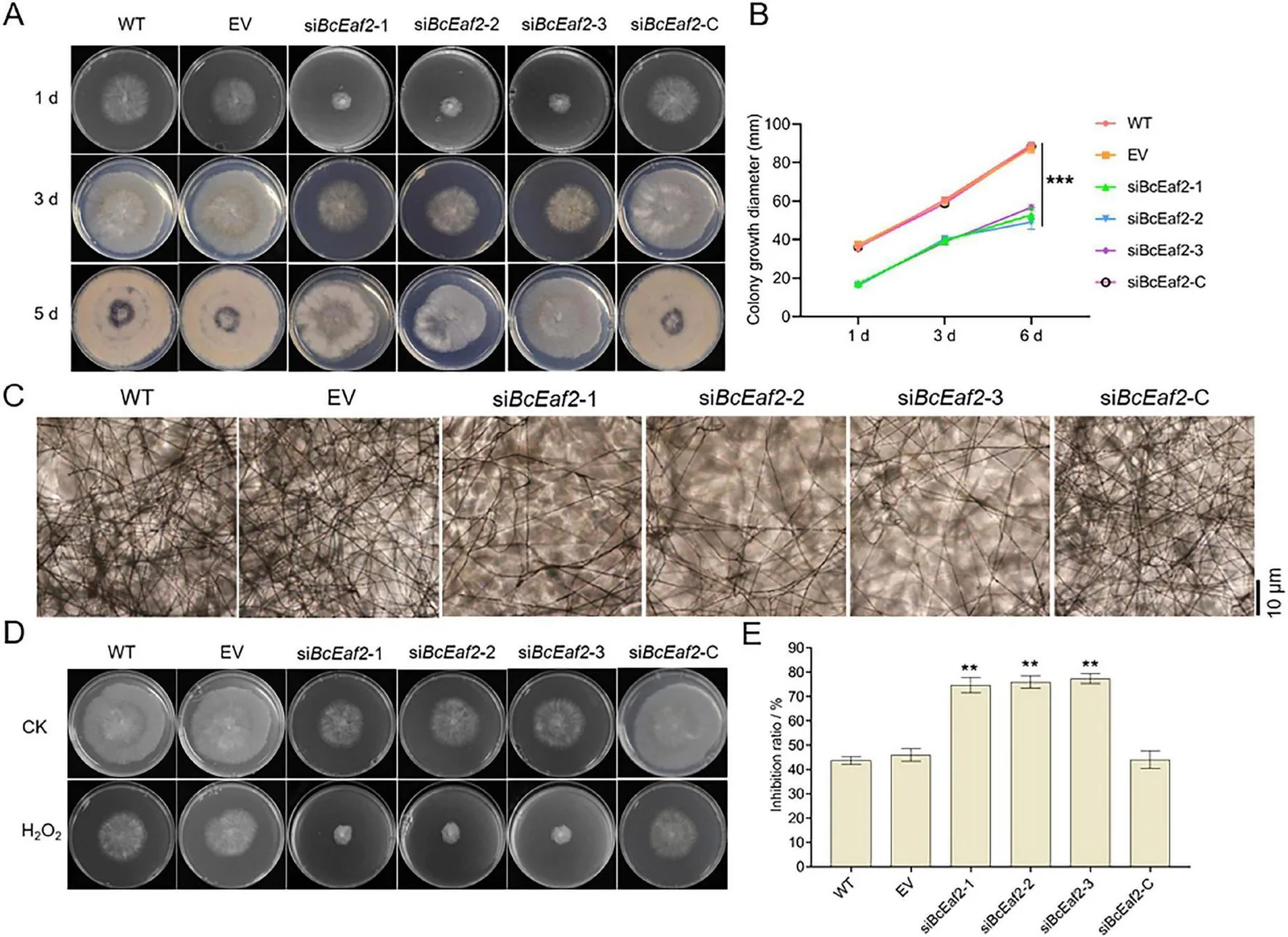
*BcEaf2* silencing impairs mycelial growth and H_2_O_2_ tolerance in *B. cinerea*. Mycelial growth of the WT (B05.10), empty vector (EV), and *BsEaf2*-silenced (siBcEaf2-1, siBcEaf2-2, and siBcEaf2-3), and the corresponding silent compensatory (siBcEaf-C) strains incubated for 3 days under different growth conditions. **(A)** The colony morphology of the above strains on PDA plates after 1 d, 3 d and 6 d of growth. **(B)** Statistical data on the growth diameter of mycelium. **(C)** Observation of the mycelial morphology of the above-mentioned strains under optical microscope. Scale bar: 10 μm. **(D)** The colony morphology of the above strains after 3 days of growth on PDA plates containing 10 mM H_2_O_2_. **(E)** Mycelial inhibition by the 10 mM H_2_O_2_ relative to untreated control was calculated. Data in panels **(B,E)** are presented as the mean ± standard deviation from three biological replicates. Statistical significance was determined according to Student’s *t*-test (**P < 0.01, ***P < 0.001).

To investigate the potential involvement of BcEaf2 in stress responses, including reactive oxygen species (ROS), and osmotic stress, we cultivated strains WT, EV, siBcEaf2-1, siBcEaf2-2, and siBcEaf2-3 in PDA medium supplemented with various stress-inducing agents. We assessed their ROS tolerance using hydrogen peroxide (H_2_O_2_) and evaluated their ability to adapt to osmotic stress through exposure to potassium chloride (KCl) and sorbitol. BcEaf2-silenced strains exhibited increased sensitivity to treatments with H_2_O_2_, KCl, and sorbitol compared to the WT and EV lines (Figures 2D,E, Supplementary Figures 2A,B). These results indicate that BcEaf2 primarily plays a role in oxidative stress and osmotic pressure processes.

### 2.3 BcEaf2 is indispensable for conidiation and pathogenicity in *B. cinerea*

In contrast to the WT and EV controls, which displayed robust and characteristic conidiation, all three *BcEaf2*-silenced strains (siBcEaf2-1, siBcEaf2-2 and siBcEaf2-3) showed a marked decrease in spore production (Figures 3A,B). Consistently, germ tube emergence occurred in more than 60% of cystospores in WT and EV strains, whereas fewer than 20% of spores from the silenced strains successfully initiated germination (Figures 3A,C). Consistent with the mycelial growth phenotype, time-course analysis at 7 and 11 days showed that the *BcEaf2*-silenced strains exhibited a persistent reduction in conidial production compared with the wild-type, EV, and complemented strains. The complemented strain largely restored conidiation to the wild-type level (Supplementary Figure 2C).

**FIGURE 3:**
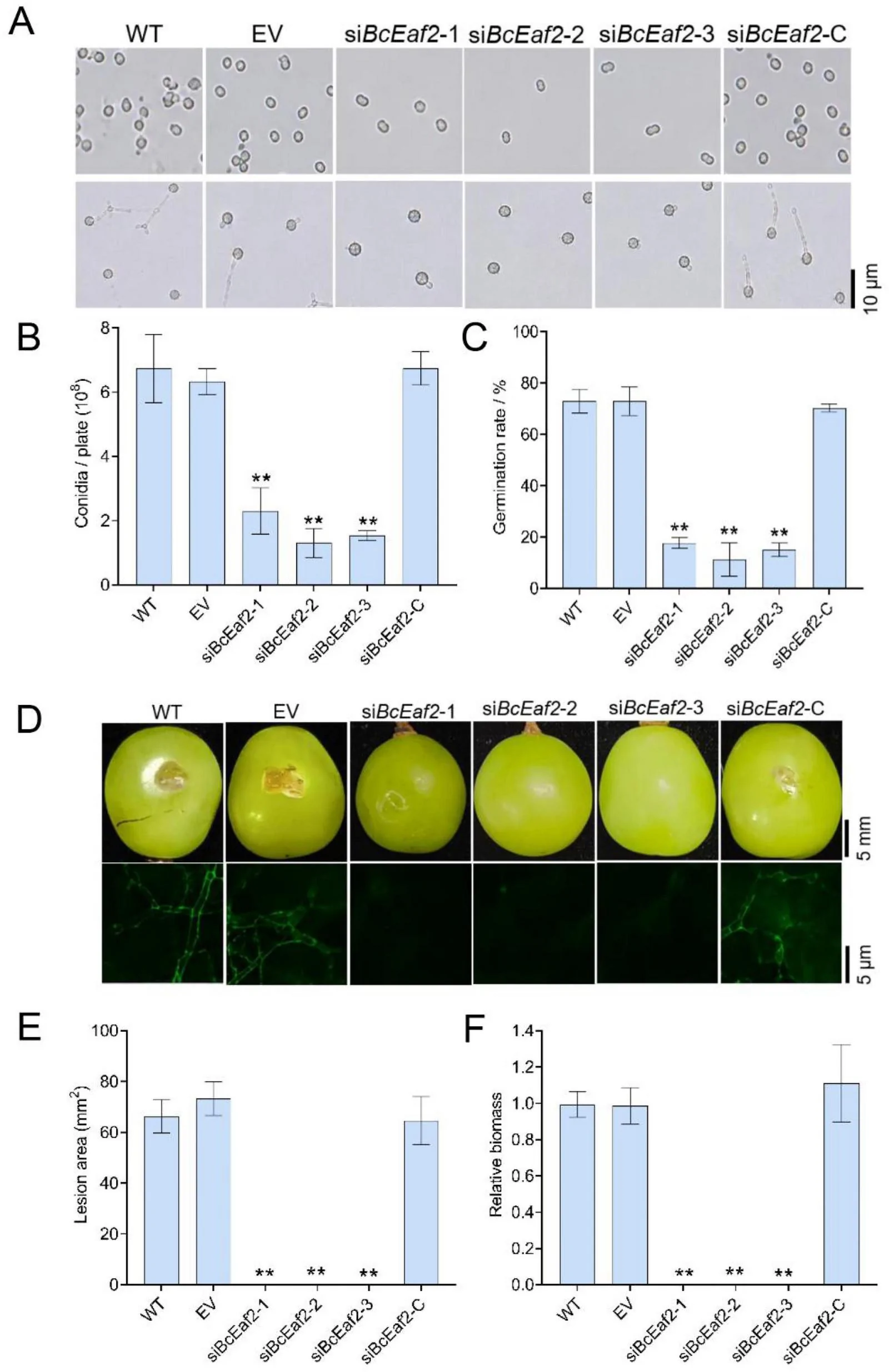
Role of the *BcEaf2* gene in asexual stage and virulence. **(A)** Microscopic observation of conidia produced by the WT (B05.10), empty vector (EV), and *BsEaf2*-silenced (siBcEaf2-1, siBcEaf2-2, and siBcEaf2-3) strains. Conidia were collected from 7-days-old cultures and observed under a light microscope. Representative images showing the morphology and production of conidia are presented (upper panel). Analysis of conidial germination. Equal numbers of conidia from the indicated strains were incubated under germination-inducing conditions, and germination was observed at the indicated time points using a light microscope. Representative images showing germination morphology and germ tube emergence are presented (down panel). Scale bars, 10 μm. **(B)** Conidia production. **(C)** Conidia germination rate. **(D)** Disease symptoms on grape tissues at 72 h post inoculation (hpi) of etiolated hypocotyls with mycelial block. Invasive hyphal growth in infected plant tissues was visualized by staining with WGA-Alexa Fluor 488 and observing under confocal laser scanning microscopy (CLSM). Representative CLSM images showing invasive hyphal colonization by the wild-type (WT), empty vector control (EV), *BcEaf2*-silenced strains, and complemented strain (siBcEaf2-C) are presented. Extensive invasive hyphal expansion and successful colonization of host tissues were observed in the WT, EV, and complemented strains, whereas *BcEaf2*-silenced strains exhibited restricted invasive growth with limited hyphal extension beyond the initial infection sites. WGA-Alexa Fluor 488 signals indicate fungal hyphal structures within infected tissues (down panel). Scale bars, 5 μm. **(E)** The area of the lesion. **(F)** Relative pathogen biomass in inoculated etiolated hypocotyls. Relative amounts of *B. cinerea* and grape DNA detected 72 hpi using the relative biomass of WT strain for normalization. **Indicates significance at *P* < 0.01. Data in panels **(B,C,E,F)** are presented as the mean ± standard deviation from three biological replicates. Statistical significance of above strains compared to the WT was determined using unpaired two-tailed *t*-tests (**P < 0.01).

The pronounced induction of BcEaf2 expression during host invasion and colonization further suggests a key role in the infection process (Figure 1C). In line with this, the silenced strains produced substantially attenuated disease symptoms on grape tissues compared with WT and EV controls (Figures 3D,E). To examine invasive hyphal development during infection, infected plant tissues were stained with WGA-Alexa Fluor 488 and observed by confocal laser scanning microscopy (CLSM). The wild-type, EV, and complemented strains exhibited extensive invasive hyphal growth and successful colonization of host tissues. In contrast, the BcEaf2-silenced strains displayed markedly restricted invasive growth, with invasive hyphae rarely extending beyond the initial infection site. These observations further demonstrate that BcEaf2 is required for normal invasive hyphal expansion and pathogenic development (Figure 3D). Quantification of fungal biomass in infected grapes revealed significantly reduced pathogen accumulation in tissues inoculated with siBcEaf2 mutants relative to those infected by WT or EV strains (Figure 3F). Together, these data establish BcEaf2 as a critical determinant of both conidiation and virulence in *Botrytis cinerea*.

### 2.4 BcEaf2 regulates histone lysine methylation H3K36me3

Initially, DMAP1 was characterized as a key cofactor of the 5mC methyltransferase DNMT1, and its depletion was shown to compromise DNMT1-mediated DNA methylation, resulting in a global reduction in 5mC levels (Lin et al., 2021; Wang et al., 2020). In addition to this role, DMAP1 harbors a conserved SANT domain, a structural module widely implicated in chromatin-associated processes through mediating interactions with histone tails, DNA, and chromatin-modifying enzymes. These observations prompted us to examine whether its fungal homolog, *BcEaf2*, exerts broader regulatory effects on chromatin states in *B. cinerea*.

To determine whether BcEaf2 broadly affects epigenetic modifications, we examined both activating and repressive histone marks in *B. cinerea* strains cultured under *in vitro* mycelial growth conditions. Specifically, we analyzed representative histone acetylation marks, including H3K18ac, H3K27ac, and H4K5ac, as well as trimethylation marks of histone H3 at lysine 4 (H3K4me3), lysine 27 (H3K27me3), and lysine 36 (H3K36me3). Silencing of *BcEaf2* did not significantly alter the global abundance of the examined histone acetylation marks, including H3K18ac, H3K27ac, and H4K5ac, compared with the wild-type strain. Similarly, no obvious changes were detected in H3K4me3 or H3K27me3 levels. In contrast, H3K36me3 abundance was consistently and markedly reduced in all *BcEaf2*-silenced strains (Figure 4), indicating a selective effect of BcEaf2 on this specific histone modification.

**FIGURE 4:**
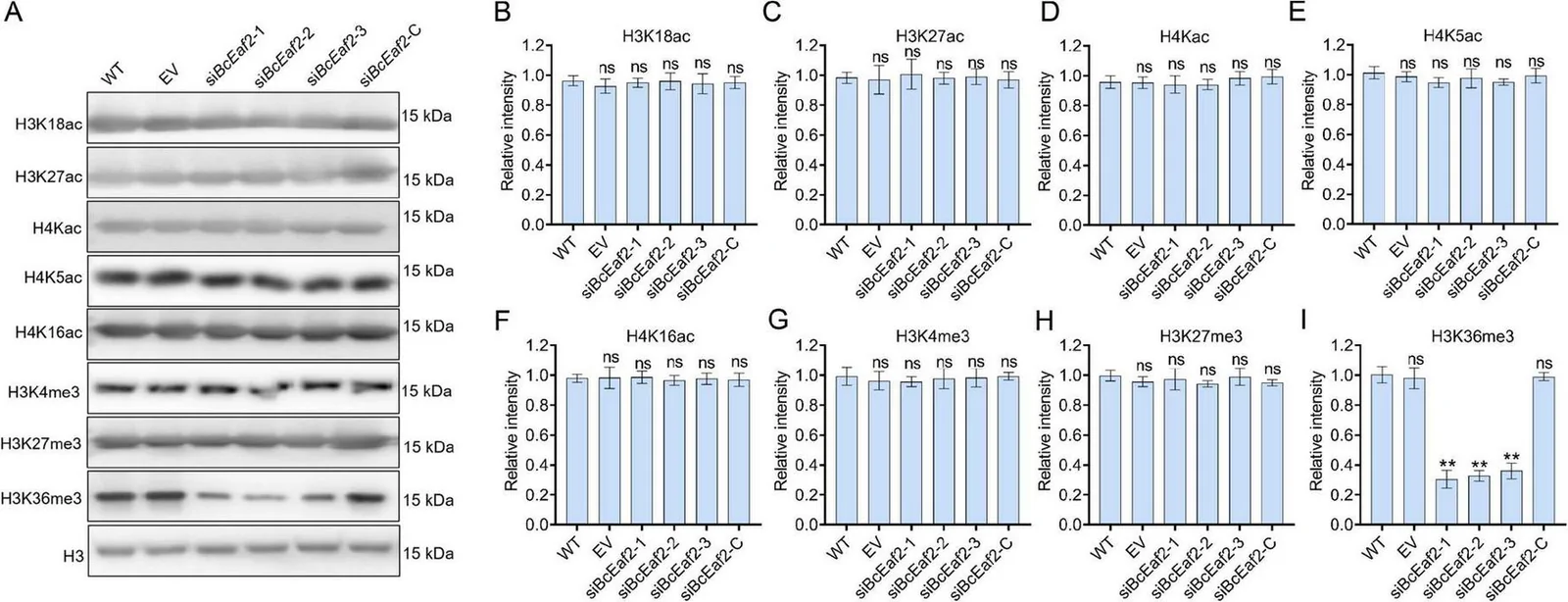
Detection of histone acetylation and methylation levels. **(A)** Western blot analysis of histone acetylation and methylation levels in the WT, siBcEaf2-1, siBcEaf2-2, and siBcEaf2-3 transformants using antibodies specific for H3K18ac, H3K27ac, H4Kac, H4K5ac, H3K4me3, H3K27me3, and H3K36me3. Histone H3 was used as a loading control. **(B–I)** Relative band intensities were quantified based on signal density using ImageJ (**P < 0.01; ns, not significant). Data in panels **(B–I)** are presented as the mean ± standard deviation (SD) from three independent biological replicates (*n* = 3). Statistical significance was determined using Student’s *t*-test for comparisons between two groups or one-way ANOVA for multiple-group comparisons. Different letters indicate significant differences among groups determined by one-way ANOVA.

### 2.5 BcEaf2 interacting with H3K36me3 methyltransferase BcSET2

To delineate the mechanism by which BcEaf2 influences H3K36me3 deposition, we performed a yeast two-hybrid (Y2H) library screen using BcEaf2 as bait. This screen recovered a candidate interactor corresponding to a homolog of the canonical H3K36me3 methyltransferase SET2, hereafter designated BcSET2 (Supplementary Table 1). Domain analysis revealed that BcSET2 retains the conserved SET catalytic domain characteristic of lysine methyltransferases, suggesting functional conservation. We therefore hypothesized that BcEaf2 may regulate H3K36me3 dynamics through association with BcSET2. Targeted Y2H assays confirmed the interaction between BcEaf2 and BcSET2 in a pairwise context, and co-immunoprecipitation (Co-IP) experiments further validated that BcEaf2 and BcSET2 form a complex *in vivo* (Figures 5A,B), supporting the physiological relevance of this interaction. To further determine the region of BcEaf2 required for BcSET2 association, a series of BcEaf2 constructs lacking the N-terminal region, SANT domain, or C-terminal region were generated and analyzed using Y2H assays. Full-length BcEaf2 exhibited a strong interaction with BcSET2, whereas deletion of the conserved SANT domain completely abolished this interaction. In contrast, removal of the N-terminal or C-terminal regions did not significantly affect the association between BcEaf2 and BcSET2. These results indicate that the SANT domain of BcEaf2 is essential for its interaction with BcSET2 and suggest that this domain contributes critically to the formation of the BcEaf2–BcSET2 regulatory complex (Figure 5A). We further attempted to generate a BcEaf2 mutant lacking the SANT domain to directly assess its effect on H3K36me3 levels. However, despite repeated attempts, no stable SANT-domain deletion mutant was obtained. Consistent with this observation, we were also unable to obtain a BcEaf2 knockout mutant, suggesting that severe disruption of BcEaf2 function may compromise the recovery or viability of the corresponding mutants. Therefore, the direct contribution of the SANT domain to H3K36me3 regulation may not be experimentally evaluated in a SANT-domain-deficient strain.

**FIGURE 5:**
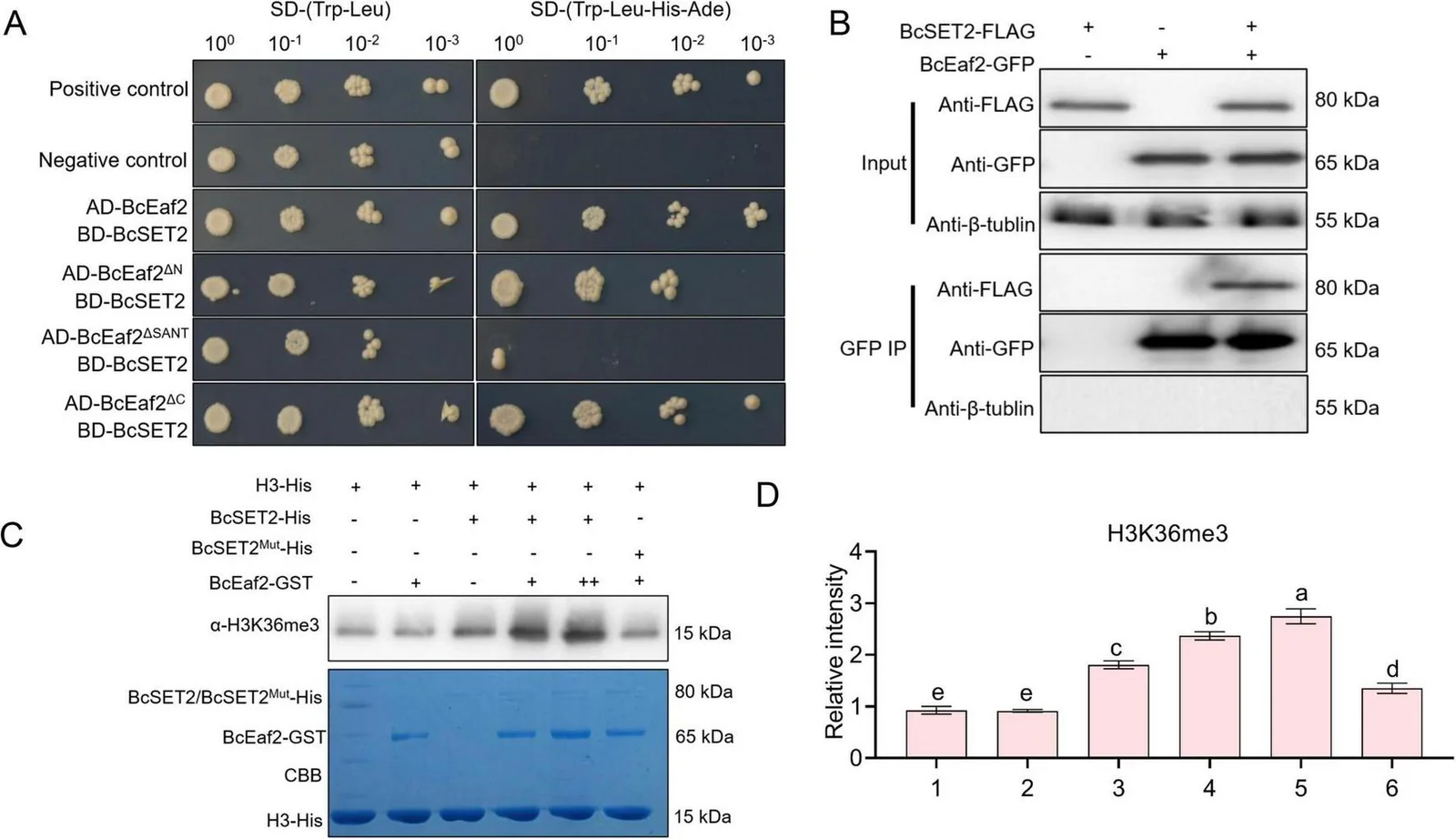
Interactions between BcEaf2 and BcSET2. **(A)** The interaction between BcEaf2 and BcSET2 was analyzed using the yeast two-hybrid (Y2H) assay. Full-length BcEaf2 and a series of BcEaf2 mutants lacking the N-terminal region (ΔN), SANT domain (ΔSANT), or C-terminal region (ΔC) were fused with the GAL4 activation domain (AD) or DNA-binding domain (BD), as indicated. Yeast cell serial dilutions were assayed for growth on selective media lacking tryptophan, leucine, histidine, and adenine. pGBKT7-Lam and pGADT7 were used as negative controls. **(B)** Co-IP assays for the interactions between BcEaf2 and BcSET2 *in vivo*. Western blots of total protein isolated from transformants expressing BcSET2-3 × FLAG and BcEaf2-GFP (input) and proteins eluted from anti-FLAG affinity beads (GFP IP) were detected with anti-FLAG and anti-GFP antibodies. **(C)** The catalytic activity of wild-type BcSET2 and a catalytic-deficient mutant (BcSET2^ Mut^) lacking the conserved NHXCXPN motif was examined *in vitro*. Protein loadings were verified by Coomassie brilliant blue (CBB) staining. **(D)** The relative intensities of the acetylated histone bands from each treatment were quantified with Image J, and lane 1 in the panel **(C)** were set as 1.00, respectively. Data in panel **(D)** are presented as the mean ± SD from three biological replicates. Different letters in panel **(D)** represent significant differences by one-way ANOVA (*P* < 0.05).

To further interrogate the functional consequence of this association, recombinant BcEaf2 and BcSET2 proteins were heterologously expressed and affinity-purified to near homogeneity. *In vitro* methyltransferase assays revealed that BcSET2 possesses intrinsic H3K36 methylation activity, consistent with its predicted role. Notably, inclusion of BcEaf2 in the reaction led to a pronounced enhancement of BcSET2-mediated H3K36me3 deposition (Figures 5C,D), indicating that BcEaf2 acts as a positive regulator of BcSET2 catalytic efficiency rather than as an independent methyltransferase. To investigate whether the promotion of H3K36 methylation by BcEaf2 is dependent on the catalytic activity of BcSET2, a catalytic-deficient mutant (BcSET2*^Mut^*) lacking the conserved NHXCXPN motif was generated (Ji et al., 2022). As expected, BcSET2*^Mut^* exhibited markedly reduced H3K36 methyltransferase activity compared with wild-type BcSET2. Notably, BcEaf2 stimulated the enzymatic activity of wild-type BcSET2 in a dose-dependent manner but had no detectable effect on BcSET2*^Mut^* (Figures 5C,D). To determine whether BcEaf2 silencing affects the expression of BcSET2, we examined BcSET2 transcript levels in the independent siBcEaf2 mutants using quantitative RT–qPCR. No significant differences in BcSET2 expression were detected between the siBcEaf2 mutants and the WT strain (Supplementary Figure 3A). These results indicate that BcEaf2 silencing does not substantially affect BcSET2 transcription under the tested conditions. To determine whether deletion of the conserved NHXCXPN motif affects BcSET2 protein abundance, we compared BcSET2 protein levels between WT and BcSET2Mut by western blot analysis. BcSET2 protein was detected at comparable levels in WT and BcSET2Mut, indicating that deletion of the NHXCXPN motif did not substantially affect BcSET2 protein expression (Supplementary Figures 3B,C). Thus, the reduced H3K36me3 level observed in BcSET2Mut is unlikely to result from altered BcSET2 protein abundance. Furthermore, to determine whether the GFP and FLAG tags affected the biological functions of BcEaf2 and BcSET2, we compared the mycelial growth and virulence of the GFP-BcEaf2 and FLAG-BcSET2 strains with those of the WT strain. No significant differences in colony growth were observed between the GFP-BcEaf2, FLAG-BcSET2, and WT strains (Supplementary Figure 4A). Similarly, the GFP-BcEaf2 and FLAG-BcSET2 strains exhibited comparable virulence to the WT strain, as indicated by lesion development on inoculated leaves (Supplementary Figure 4B). These results indicate that the GFP or FLAG tag did not substantially affect the mycelial growth or virulence of the corresponding strains. These results indicate that BcEaf2 promotes BcSET2-dependent H3K36me3 formation, although whether BcEaf2 directly affects the catalytic efficiency of BcSET2 remains to be determined.

### 2.6 BcSET2 plays a pivotal role in hyphal growth, stress responses, conidiation, and pathogenicity in *B. cinerea*

Given that the biological function of *SET2* in *B. cinerea* has not yet been characterized, we generated *BcSET2* knockout mutants (Δ*BcSET2*-1, Δ*BcSET2*-2 and Δ*BcSET2*-3) and the corresponding complemented (Δ*BcSET2*-C) strains using homologous recombination (Supplementary Figures 3D,E). Although colony morphology remained largely unchanged, all *BcSET2* knockout mutants (Δ*BcSET2*-1, Δ*BcSET2*-2 and Δ*BcSET2*-3) displayed a pronounced reduction in radial growth relative to both WT, EV controls, and Δ*BcSET2*-C (Figures 6A,B). To clarify whether *BcSET2* knockout strains exhibit delayed growth or growth arrest, we performed additional time-course analyses of mycelial growth by measuring colony diameters at 1, 3, and 6 days post-inoculation. As shown in the revised Figure 6C, the *BcSET2* knockout strains displayed a consistently and significantly reduced growth rate throughout the observation period. Notably, by 6 days, the wild-type, EV, and complemented strains had completely covered the Petri dishes, whereas the *BcSET2* knockout strains still failed to fully occupy the plates, indicating a persistent growth defect rather than a temporary developmental delay (Figure 6C). Furthermore, Microscopic observation showed that the *BcSET2* knockout strains exhibited a markedly sparser hyphal network than the wild-type, EV, and complemented strains. The hyphae were less densely distributed, with fewer branches and larger gaps between neighboring hyphae, whereas the complemented strain largely restored the wild-type phenotype (Figure 6A).

**FIGURE 6:**
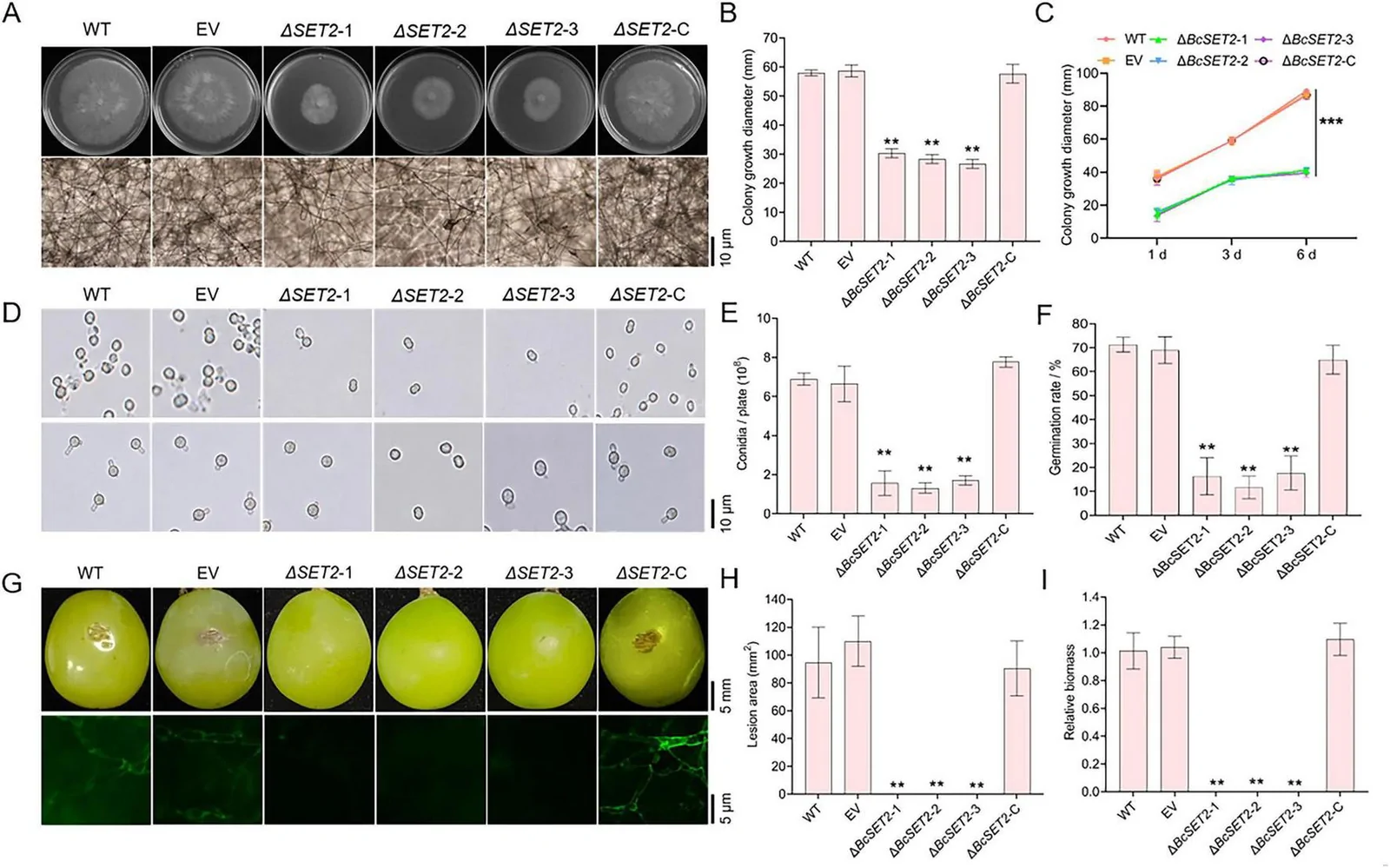
BcSET2 plays a pivotal role in hyphal growth, stress responses, conidiation, and pathogenicity in *B. cinerea*. **(A)** Mycelial growth of the WT (B05.10), empty vector (EV), and *BcSET2* knockout mutants (Δ*BcSET2*-1, Δ*BcSET2*-2 and Δ*BcSET2*-3) and the corresponding complemented (Δ*BcSET2*-C) incubated for 3 days on PDA plate (upper panel). Observation of the mycelial morphology of the above-mentioned strains under optical microscope. Scale bar: 10 μm (down panel). **(B)** The colony diameters of these strains at 3 days. **(C)** The colony diameters of the indicated strains were measured after 1, 3, and 6 days of incubation on PDA plates. The growth dynamics of the above strains were evaluated based on colony expansion over time. **(D)** Microscopic observation of conidia produced by the indicated strains. Conidia were collected from 7-days-old cultures and observed under a light microscope. Representative images showing the morphology and production of conidia are presented (upper panel). Analysis of conidial germination. Equal numbers of conidia from the indicated strains were incubated under germination-inducing conditions, and germination was observed at the indicated time points using a light microscope. Representative images showing germination morphology and germ tube emergence are presented (down panel). Scale bars, 10 μm. **(E)** Conidia production. **(F)** Conidia germination rate. **(G)** Disease symptoms on grape tissues at 72 h post inoculation (hpi) of etiolated hypocotyls with mycelial block. Invasive hyphal growth in infected plant tissues was visualized by staining with WGA-Alexa Fluor 488 and observing under confocal laser scanning microscopy (CLSM). Representative CLSM images showing invasive hyphal colonization by the above strains are presented. Extensive invasive hyphal expansion and successful colonization of host tissues were observed in the WT, EV, and complemented strains, whereas *BcEaf2*-silenced strains exhibited restricted invasive growth with limited hyphal extension beyond the initial infection sites. WGA-Alexa Fluor 488 signals indicate fungal hyphal structures within infected tissues (down panel). Scale bars, 5 μm. **(H)** The area of the lesion. **(I)** Relative pathogen biomass in inoculated etiolated hypocotyls. Relative amounts of *B. cinerea* and grape DNA detected 72 hpi using the relative biomass of WT strain for normalization. **Indicates significance at *P* < 0.01. Data in panels **(B,C,E,F,H,I)** are presented as the mean ± standard deviation from three biological replicates. Statistical significance of above strains compared to the WT was determined using unpaired two-tailed *t*-tests (**P < 0.01).

To investigate the potential involvement of BcSET2 in stress responses, including reactive oxygen species (ROS), and osmotic stress, we cultivated strains WT, EV, Δ*BcSET2*-1, Δ*BcSET2*-2, Δ*BcSET2*-3, and Δ*BcSET2*-C in PDA medium supplemented with various stress-inducing agents. We assessed their ROS tolerance using hydrogen peroxide (H_2_O_2_) and evaluated their ability to adapt to osmotic stress through exposure to potassium chloride (KCl) and sorbitol. Δ*BcSET2*-1, Δ*BcSET2*-2 and Δ*BcSET2*-3 mutants exhibited increased sensitivity to treatments with H_2_O_2_, KCl, and sorbitol compared to the WT, EV controls, and Δ*BcSET2*-C (Supplementary Figures 4C–E). These results indicate that BcSET2 primarily plays a role in nutritional growth, oxidative stress, and osmotic pressure processes of *B. cinerea.*

In contrast to the WT, EV controls, and Δ*BcSET2*-C, which displayed robust and characteristic conidiation, Δ*BcSET2*-1, Δ*BcSET2*-2 and Δ*BcSET2*-3 mutants showed a marked decrease in conidia production (Figures 6D,E). Consistently, germ tube emergence occurred in more than 60% of cystospores in WT, EV controls, and Δ*BcSET2*-C, whereas fewer than 20% of conidia from Δ*BcSET2*-1, Δ*BcSET2*-2 and Δ*BcSET2*-3 mutants successfully initiated germination (Figures 6D,F). Consistent with the mycelial growth phenotype, time-course analysis at 7 and 11 days showed that the *BcSET2* knockout strains exhibited a persistent reduction in conidial production compared with the wild-type, EV, and complemented strains. The complemented strain largely restored conidiation to the wild-type level (Supplementary Figure 4F).

In line with this, Δ*BcSET2*-1, Δ*BcSET2*-2 and Δ*BcSET2*-3 mutants produced substantially attenuated disease symptoms on grape tissues compared with WT, EV controls, and Δ*BcSET2*-C (Figures 6G,H). Consistent with the phenotype of the *BcEaf2*-silenced strains, the Δ*BcSET2* mutant also exhibited markedly restricted invasive hyphal growth *in planta*. WGA–Alexa Fluor 488 staining combined with CLSM revealed that invasive hyphae of the Δ*BcSET2* mutant were largely confined to the inoculation site and showed minimal expansion within host tissues, whereas the wild-type and complemented strains successfully colonized and spread throughout the infected tissues (Figure 6G). Quantification of fungal biomass in infected grapes revealed significantly reduced pathogen accumulation in tissues inoculated with Δ*BcSET2*-1, Δ*BcSET2*-2 and Δ*BcSET2*-3 mutants relative to those infected by WT, EV controls, and Δ*BcSET2*-C (Figure 6I). Given the reported involvement of DMAP1-related proteins in DNA damage responses, we next investigated whether BcEaf2 contributes to the response of *B. cinerea* to DNA damage stress. The sensitivity of *BcEaf2*-silenced strains to the DNA-damaging agent methyl methanesulfonate (MMC) was examined. Compared with the wild-type (WT) and empty-vector (EV) strains, all three independent *BcEaf2*-silenced strains (siBcEaf2-1, siBcEaf2-2, and siBcEaf2-3) exhibited significantly increased sensitivity to MMC, as indicated by reduced mycelial growth on MMC-containing medium (Supplementary Figures 5A,B). Complementation of *BcEaf2* substantially restored MMC tolerance in the siBcEaf2-C strain. We next examined whether BcSET2 contributes to the same stress response. Similarly, three independent *BcSET2* knockout strains (Δ*BcSET2*-1, Δ*BcSET2*-2, and Δ*BcSET2*-3) displayed increased sensitivity to MMC compared with the WT and EV strains, whereas complementation of *BcSET2* restored MMC tolerance (Supplementary Figures 5A,B). These results indicate that both BcEaf2 and BcSET2 contribute to the tolerance of *B. cinerea* to DNA damage stress. Together, these data establish BcSET2 plays a pivotal role in hyphal growth, stress responses, conidiation, and pathogenicity *in B. cinerea.*

## 3 Discussion and conclusions

Histone modifications constitute a central regulatory layer linking chromatin dynamics to fungal development and pathogenicity (Doyon et al., 2004; Ji et al., 2022; Mohan et al., 2011; Penicud and Behrens, 2014). In this study, we identify BcEaf2 as an essential regulator of growth, stress adaptation and virulence in *B. cinerea*, and uncover an unexpected epigenetic mechanism whereby BcEaf2 modulates H3K36me3 through functional coupling with histone methyltransferase *BcSET2*. These findings extend the functional repertoire of Eaf2/DMAP1 family proteins and reveal a mode of chromatin regulation in a major phytopathogenic fungus.

Eaf2/DMAP1 proteins have been primarily characterized in animals and yeast as components of chromatin-associated complexes. In mammals, DMAP1 was originally identified as a DNMT1-associated factor required for maintenance DNA methylation and genome stability (Doyon et al., 2004; Penicud and Behrens, 2014). In yeast, the Eaf2 ortholog functions as a subunit of the NuA4 histone acetyltransferase complex, contributing predominantly to histone H4 acetylation and transcriptional activation (Ji et al., 2022; Krogan et al., 2004; Li et al., 2024; Mohan et al., 2011). In contrast to these established roles, our data demonstrate that BcEaf2 does not measurably influence global H4 acetylation but instead selectively regulates H3K36me3 levels. This divergence suggests that the molecular function of Eaf2 has undergone substantial evolutionary rewiring, potentially reflecting lineage-specific chromatin architectures and regulatory demands in filamentous fungi.

H3K36me3 is a highly conserved histone mark associated with transcription elongation, RNA processing, and genome integrity (Chen et al., 2025; Markert et al., 2025; Michail et al., 2025). In yeast and metazoans, SET2 is the sole methyltransferase responsible for H3K36 trimethylation and is recruited co-transcriptionally via interaction with elongating RNA polymerase II (Boulanger et al., 2024; Lee et al., 2025; Lim et al., 2024). Consistent with this paradigm, we demonstrate that BcSET2 is indispensable for H3K36me3 deposition and plays critical roles in vegetative growth, stress tolerance, conidiation and pathogenicity. These phenotypes mirror those reported in other fungal pathogens (Xu et al., 2023). Recent studies across diverse filamentous pathogens, including *Aspergillus flavus*, *Metarhizium robertsii*, and the oomycete *Phytophthora sojae*, have demonstrated that histone lysine methyltransferases regulate fungal gene expression by modulating H3K36 methylation, thereby influencing development and host infection. In *A. flavus*, members of the SET2 histone methyltransferase family, such as AshA and SetB, have been shown to participate in morphogenesis and secondary metabolism by regulating key transcription factors. These enzymes are essential for aflatoxin biosynthesis and play critical roles in fungal toxicity toward both crops and animals (Zhuang et al., 2022). Studies in entomopathogenic fungi further reveal that deletion of the histone lysine methyltransferase gene MrASH1 in *M. robertsii* leads to a pronounced defect in host cuticle penetration. This phenotype arises from impaired lipid droplet mobilization within appressoria, resulting in reduced glycerol production and insufficient turgor pressure for successful penetration. Western blot analyses indicate that MrASH1 catalyzes H3K36me2 modification. Moreover, integrated RNA-seq and ChIP-seq analyses demonstrate that MrASH1 activates the expression of the peroxisome-associated gene Mrpex16 via H3K36me2 deposition. Consistently, deletion of Mrpex16 recapitulates the phenotype of the mrash1 mutant, highlighting a direct regulatory pathway linking H3K36 methylation to infection-related development (Wang et al., 2023). In oomycete plant pathogens, the expression of numerous effector genes is dynamically reprogrammed during infection, yet the underlying regulatory mechanisms remain poorly understood. A recent study identified a SET domain–containing protein, PsKMT3, in *P. sojae*, which is strongly induced at early stages of infection. Disruption of PsKMT3 results in severe defects in asexual development and pathogenicity. Genome-wide chromatin profiling and immunoblot analyses revealed a marked reduction in H3K36me3 levels in the mutant, indicating that this modification plays a pivotal role in regulating infection-associated transcriptional programs (Chen H. et al., 2023). Collectively, these findings underscore the conserved importance of H3K36 methylation and its associated methyltransferases in governing proliferation, virulence, and pathogenicity in filamentous pathogens. A deeper mechanistic understanding of H3K36 methylation dynamics and its regulatory machinery will not only advance our knowledge of fungal epigenetics but also facilitate the development of novel antifungal strategies and disease management approaches.

A central advance of this study is the identification of BcEaf2 as a positive regulator of BcSET2 activity. Through protein-protein interaction assays and *in vitro* reconstitution, we demonstrate that BcEaf2 directly associates with BcSET2 and markedly enhances its catalytic efficiency toward H3K36 methylation. This finding uncovers a previously unappreciated regulatory layer controlling SET2-dependent histone methylation. Although SET domain–containing methyltransferases are known to be modulated by multiprotein complex assembly, post-translational modifications, and chromatin context (Nikolopoulos et al., 2025; Shen Y. et al., 2025), direct stimulation of SET2 enzymatic activity by accessory cofactors has remained largely unexplored. Our data raise the possibility that *BcEaf2* acts as a functional cofactor that fine-tunes BcSET2 activity at multiple levels. One plausible model is that *BcEaf2* stabilizes BcSET2 protein conformation, thereby enhancing its catalytic competence. Alternatively, *BcEaf2* may facilitate substrate recognition by promoting the interaction of BcSET2 with nucleosomal histone H3, potentially through its conserved SANT domain, which is known to mediate chromatin engagement (Chandrasekaran et al., 2024; Shen W. et al., 2025; Wanat et al., 2025). Another possibility is that BcEaf2 promotes an active enzymatic conformation of BcSET2, analogous to regulatory subunits described for other chromatin-modifying complexes. In addition, BcEaf2 may contribute to the recruitment or retention of BcSET2 at specific genomic loci, thereby linking enzymatic activity to transcriptional context. Importantly, this regulatory mode may provide a mechanism for dynamic and context-dependent control of H3K36me3 deposition during fungal development and host infection. Given that H3K36me3 is tightly coupled to transcription elongation, modulation of BcSET2 activity by BcEaf2 could enable rapid reprogramming of gene expression in response to environmental or host-derived cues. This is particularly relevant for phytopathogenic fungi such as *B. cinerea*, which must swiftly adapt to fluctuating oxidative stress and nutrient conditions during infection. Beyond its implications in fungal biology, the BcEaf2*-*BcSET2 module may reflect a broader and previously underappreciated paradigm in epigenetic regulation, whereby non-enzymatic cofactors directly potentiate the activity of histone methyltransferases. Future studies integrating structural biology, chromatin immunoprecipitation sequencing (ChIP-seq), and proteomics will be essential to dissect the precise molecular mechanism underlying this interaction (Belviso et al., 2024; Kim et al., 2023; Maas et al., 2024; Zhang et al., 2024). In particular, resolving the structure of the BcEaf2*-*BcSET2 complex and mapping their genomic co-occupancy will provide critical insights into how catalytic enhancement is coupled to chromatin targeting and transcriptional regulation.

The phenotypic parallels between BcEaf2-silenced strains and BcSET2 deletion mutants further support a functional epistasis between these two factors. Both perturbations lead to impaired hyphal growth, heightened sensitivity to oxidative and osmotic stresses, reduced conidiation, and attenuated virulence. Given that H3K36me3 is enriched within actively transcribed gene bodies, its reduction is likely to compromise transcriptional programs required for stress adaptation and infection-related development. Indeed, oxidative stress tolerance and host colonization in *B. cinerea* are tightly linked to transcriptional reprogramming and ROS detoxification pathways (de la Osa et al., 2025; Ma et al., 2024; Yalcin et al., 2024). It is therefore plausible that the BcEaf2-BcSET2 axis regulates a subset of genes critical for these processes.

The essentiality of *BcEaf2*, as inferred from the repeated failure to obtain null mutants, underscores its fundamental role in the viability and cellular homeostasis of *B. cinerea*. This observation is consistent with the embryonic lethality reported for loss of DMAP1 in mammals (Doyon et al., 2004; Xin et al., 2004), suggesting that members of the Eaf2/DMAP1 family fulfill indispensable functions in genome regulation across eukaryotic lineages. Despite this apparent conservation at the level of essentiality, our findings point to a striking divergence in molecular function. Whereas DMAP1 in mammals primarily participates in DNA methylation maintenance through association with DNMT1 and chromatin-modifying complexes, BcEaf2 appears to operate predominantly within the histone methylation landscape, specifically influencing H3K36me3 deposition. This functional shift highlights the remarkable plasticity of chromatin regulatory networks, whereby conserved protein scaffolds can be repurposed to engage distinct epigenetic pathways in different organisms. One plausible explanation is that BcEaf2 has evolved to compensate for lineage-specific differences in epigenomic architecture, such as reduced reliance on DNA methylation and increased dependence on histone-based regulation in filamentous fungi. In this context, BcEaf2 may act as a critical integrator of chromatin signals, coordinating transcriptional programs required for growth, stress adaptation, and pathogenicity. Moreover, the essential nature of BcEaf2 raises the possibility that it regulates a core set of housekeeping genes or chromatin processes that are indispensable for fungal survival. Its impact on H3K36me3, a modification tightly linked to transcriptional elongation and genome stability, further supports this notion (Han et al., 2025). Disruption of such a central regulatory node would therefore be expected to have pleiotropic consequences, consistent with the broad defects observed in growth and stress responses. From an evolutionary perspective, these findings suggest that while the Eaf2/DMAP1 protein family retains a conserved role in maintaining chromatin integrity, the specific epigenetic mechanisms through which this function is executed have diversified. This divergence likely reflects adaptation to distinct genomic and ecological contexts, reinforcing the concept that epigenetic regulators are both conserved and highly adaptable components of eukaryotic biology (Cawood and Ton, 2025; Cohen et al., 2025; Madhani, 2025; Sommer, 2025).

Eaf2/DMAP1 is a conserved chromatin-associated factor with functions extending beyond a single type of histone modification. In *Saccharomyces cerevisiae*, Eaf2/Swc4 is a component of the NuA4 and SWR1 chromatin regulatory complexes and contributes to histone acetylation and chromatin regulation. In mammals, DMAP1 has also been reported to associate with DNMT1 and stimulate DNA methylation, in addition to its association with the TIP60-p400 histone acetyltransferase complex and its role in DNA damage-responsive H4K16 acetylation (Lee et al., 2010; Rountree et al., 2000). Thus, the conserved Eaf2/DMAP1 family is not restricted to the regulation of histone acetylation but participates in multiple layers of epigenetic and chromatin regulation. In this context, our finding that BcEaf2 is associated with BcSET2-dependent H3K36 methylation does not necessarily represent a functional replacement of an acetylation-related activity, but may instead reflect functional diversification of a conserved chromatin regulator. H3K36 methylation is broadly conserved in fungi, whereas the regulatory networks controlling this modification can differ among fungal lineages. Differences in protein–protein interactions, the composition of associated chromatin-modifying complexes, and local chromatin environments may therefore allow conserved Eaf2/DMAP1 proteins to engage distinct epigenetic pathways in different species. Our identification of a BcEaf2–BcSET2–H3K36me3 regulatory axis suggests that BcEaf2 may have acquired or retained a species-context-dependent connection to H3K36 methylation in *B. cinerea*, while maintaining its broader conserved role in chromatin regulation. Whether this functional association is specific to *B. cinerea* or represents a more broadly conserved feature among filamentous fungi will require comparative analyses across additional fungal species.

From an applied perspective, the BcEaf2–BcSET2 regulatory module may represent a potential target for antifungal intervention, given its important roles in fungal growth, development, stress responses, and pathogenicity. In addition to conventional chemical approaches, RNA interference (RNAi)-based strategies, including spray-induced gene silencing (SIGS) or nucleic acid-based fungicides, may provide alternative approaches to interfere with the expression or function of BcEaf2 or BcSET2. However, the feasibility and efficacy of targeting this regulatory module through such approaches remain to be experimentally evaluated. In summary, we propose a model in which BcEaf2 acts as a epigenetic regulator that fine-tunes H3K36me3 deposition by directly modulating BcSET2 activity. This mechanism adds an additional regulatory layer to the control of transcription-associated chromatin modifications and provides new insight into how epigenetic processes coordinate fungal development and pathogenicity. Given the importance of *B. cinerea* as a globally destructive plant pathogen, targeting such chromatin regulatory pathways may offer novel strategies for disease control.

## 4 Materials and methods

### 4.1 Strains and culture conditions

The *Botrytis cinerea* strain B05.10 used in this study was kindly provided by Professor Matthias Hahn (University of Kaiserslautern, Germany). Fungal strains were routinely cultured on potato dextrose agar (PDA) at 25 °C. Under continuous light conditions, conidiation was induced and spores were harvested after 7 days. Conidia were collected in sterile distilled water and quantified using a hemocytometer. Following enumeration, the spore suspension was centrifuged at 4,500 *g* for 10 min, and the resulting pellet was resuspended in potato dextrose broth (PDB). The final spore concentration was adjusted to 2 × 10^8^ conidia mL^−1^ for subsequent germination assays.

### 4.2 Identification of BcEaf2 and BcSET2 in *B. cinerea*

Based on the *B. cinerea* genome database^1^, the *Eaf2* and *SET2* protein sequences from *Saccharomyces cerevisiae* were used as queries to identify the corresponding homologs, *BcEaf2* and *BcSET2*, through BLASTP analysis. To determine the presence and length of introns, total RNA was extracted from mycelia of the parental strain B05.10 using the RNAsimple Total RNA Kit (Tiangen Biotech, Beijing, China). First-strand cDNA was subsequently synthesized using the PrimeScript RT Reagent Kit (TaKaRa, Dalian, China). The resulting cDNA was then used as a template for PCR amplification with primer pair P1/P2 (primer sequences are listed in Supplementary Table 2). The amplified products were purified, cloned, and subjected to sequencing analysis.

### 4.3 Generation of mutants and complemented strains

To disrupt the target gene BcSET2 in the B05.10 strain, a polyethylene glycol (PEG)-mediated protoplast transformation approach was employed (Han et al., 2025). Putative transformants were screened by diagnostic PCR using specific primer pairs listed in Supplementary Table 2 (corresponding electrophoresis results are shown in Supplementary Figure 2). Primer pair F1/R1 was used to verify correct replacement of the target locus via homologous recombination, whereas primer pair F2/R2 was designed to confirm complete deletion of the BcSET2 gene (primer sequences are listed in Supplementary Table 2).

### 4.4 RNA extraction and detection of gene expression

RNA extraction from B05.10 strain and transformants followed established protocols (Heller and Tudzynski, 2011). RNA integrity was evaluated through agarose gel electrophoresis. Complementary DNA was synthesized from 1 to 5 μg of total RNA using oligonucleotide primers and a reverse transcriptase kit (AH341; TransGen Biotech), following the protocol of manufacturer.

Quantitative real-time PCR (qRT-PCR) was performed using SYBR™ Green Universal Master Mix (Thermo Fisher Scientific, Waltham, MA, USA) in a 20 μL reaction mixture containing 10 μL of SYBR™ Green Universal Master Mix, 0.4 μM of each gene-specific primer, 2 μL of diluted cDNA template, and nuclease-free water. The qRT-PCR amplification was performed using the following thermal cycling conditions: initial denaturation at 95 °C for 10 min, followed by 40 cycles of denaturation at 95 °C for 15 s, annealing/extension at 60 °C for 40 s. A melting curve analysis was subsequently performed to verify the specificity of PCR amplification. Relative transcript abundance was calculated using the 2^–ΔΔCt^ method, with *tubulin* used as the endogenous reference gene. Three biological replicates were analyzed for each sample, and each reaction was performed with technical replicates.

### 4.5 Stress testing

To assess the responses of *B. cinerea* to environmental stresses, all strains were inoculated onto solid media supplemented with specific stress-inducing agents. For osmotic stress assays, mycelial plugs (5 mm in diameter) were inoculated onto PDA plates supplemented with either 0.5 M KCl or 0.5 M sorbitol. The plates were incubated at 25 °C under continuous darkness, and colony diameters were measured after the indicated incubation period. For oxidative stress assays, strains were cultured on PDA agar supplemented with 10 mM H_2_O_2_ and incubated at 25 °C in the dark. Control plates without stress agents were included for comparison. Colony diameter was measured along two perpendicular axes after 5 days of incubation, and the average value was calculated for each colony. Growth inhibition rates were determined relative to the corresponding control conditions. All experiments were conducted with at least three independent biological replicates, each including three technical repeats, to ensure reproducibility and statistical robustness.

### 4.6 Virulence examination

#### 4.6.1 Pathogenicity assay on grape berries

Pathogenicity of *B. cinerea* strains was assessed using a mycelial plug inoculation assay on detached grape berries (*Vitis vinifera* cv. “Shine Muscat”). Healthy berries of uniform size and maturity were surface-sterilized by immersion in 75% ethanol for 30 s, followed by 1% sodium hypochlorite for 2 min, rinsed three times with sterile distilled water, and air-dried under sterile conditions. Each berry was gently wounded at the equatorial region with a sterile needle (approximately 3 mm deep) prior to inoculation. Mycelial plugs (5 mm in diameter) were excised from the actively growing margins of 3-days-old PDA cultures and placed onto the wound sites, while sterile PDA plugs were used as controls. Inoculated fruits were incubated at 22 °C–25 °C under high humidity (>90%) in the dark. Lesion development was monitored, and lesion diameters were measured at 3 days post-inoculation. Each treatment included at least three independent biological replicates with multiple fruits per replicate. For fungal biomass quantification, infected grape tissues were collected, and genomic DNA was extracted using the Plant Genomic DNA Kit (DP305, Tiangen Biotech, Beijing, China) according to the manufacturer’s instructions. The extracted genomic DNA was subsequently used as the template for quantitative PCR analysis with *B. cinerea*-specific primers to determine fungal biomass.

### 4.7 Co-immunoprecipitation (Co-IP) assays

For co-immunoprecipitation assays, *B. cinerea* transformants co-expressing GFP-tagged BcEaf2 and FLAG-tagged BcSET2 were generated by protoplast-mediated transformation. The GFP-BcEaf2 and FLAG-BcSET2 expression constructs were co-transformed into the wild-type strain, and transformants were selected on hygromycin-containing PDA medium. Positive transformants were confirmed by western blot prior to Co-IP experiments. *In vivo* protein–protein interactions were examined using *B. cinerea* transformants co-expressing FLAG-tagged BcSET2 and GFP-tagged BcEaf2. Mycelia were harvested and ground into a fine powder in liquid nitrogen, and total proteins were extracted using an extraction buffer containing 50 mM Tris-HCl (pH 7.5), 150 mM NaCl, 10% (v/v) glycerol, 1 mM EDTA, 1% (v/v) Triton X-100, and 1× protease inhibitor cocktail. After centrifugation, the clarified supernatant was collected and incubated with 25 μL of GFP-Trap_A agarose beads (Chromotek, Planegg-Martinsried, Germany) for 3–4 h at 4 °C to capture GFP-tagged protein complexes. The beads were then pelleted and washed three times with wash buffer containing 50 mM Tris-HCl (pH 7.5), 150 mM NaCl, and 0.1% (v/v) Triton X-100 to remove non-specifically bound proteins. Bound proteins were eluted by boiling the beads for 5 min in 2× SDS loading buffer (125 mM Tris-HCl, pH 6.8, 4% SDS, 20% glycerol, 0.02% bromophenol blue, and 10% β-mercaptoethanol) and subsequently analyzed by immunoblotting using an anti-FLAG M2 horseradish peroxidase (HRP)-conjugated antibody (EasyBio, BE2004; 1:5000 dilution) to detect FLAG-tagged BcSET2. Where applicable, HRP-conjugated secondary antibodies (goat anti-rabbit IgG or goat anti-mouse IgG; 1:5000 dilution; Beijing Lablead Biotech) were used. Protein signals were detected using the Omni-ECL™ Ultra-Sensitive Chemiluminescence Detection Kit (fg level) (Yamei Biology, Shanghai, China) and images were captured with an ImageQuant LAS4000 mini system (GE Healthcare, Chicago, USA).

### 4.8 Y2H assays

Yeast two-hybrid (Y2H) assays were conducted using the BD Matchmaker Library Construction and Screening Kit (Clontech, Palo Alto, CA) following the manufacturer’s guidelines. The full-length open reading frames (ORFs) of candidate genes were amplified from *B. cinerea* cDNA and inserted into the bait vector pGBKT7 and the prey vector pGADT7. All constructs were verified by DNA sequencing to ensure correct insertion and reading frame prior to use. The bait and prey plasmids were co-transformed into *Saccharomyces cerevisiae* strain Y2H Gold using the lithium acetate/single-stranded carrier DNA/PEG method. As controls, pGBKT7-53 combined with pGADT7 was used as a positive control, whereas pGBKT7-LAM paired with pGADT7 served as a negative control. Transformants were initially selected on SD medium lacking tryptophan and leucine (SD/-Trp/-Leu), and subsequently transferred to more stringent selective media lacking tryptophan, leucine, and histidine (SD/-Trp/-Leu/-His), or lacking tryptophan, leucine, histidine, and adenine (SD/-Trp/-Leu/-His/-Ade). Plates were incubated at 30 °C for 3 days before growth was assessed. All experiments were independently repeated at least three times to ensure reproducibility.

### 4.9 Detection of histone modification levels by western blot analysis

To determine the effects of different genetic modifications on global histone modification levels, the indicated *B. cinerea* strains were cultured in PDA liquid medium under *in vitro* conditions. Mycelial samples were harvested at the same developmental stage and used for subsequent histone modification analysis. Total proteins were extracted from fungal mycelia using protein extraction buffer, and protein concentrations were determined using a BCA protein assay kit. Equal amounts of protein samples were separated by SDS-PAGE and transferred onto PVDF membranes. The membranes were blocked with 5% skim milk and incubated with specific primary antibodies against histone modifications overnight at 4 °C. The following antibodies were used for the detection of histone modification marks: anti-H3K18ac (Abcam, ab1191), anti-H3K27ac (Abcam, ab177178), anti-H4K5ac (Abcam, ab51997), anti-H4K16ac (Abcam, ab109463), anti-H3K4me3 (Abcam, ab213224), anti-H3K27me3 (Abcam, ab192985), anti-H3K36me3 (Abcam, ab282572), and anti-total histone H3 (Abcam, ab1791). After incubation with corresponding HRP-conjugated secondary antibodies, signals were detected using an enhanced chemiluminescence (ECL) detection system. Total histone H3 was used as a loading control for normalization of histone modification levels. Relative abundance of each histone modification was quantified using ImageJ software.

### 4.10 *In vitro* H3K36me3 methyltransferase activity assay

To assess whether BcEaf2 modulates the enzymatic activity of BcSET2, an *in vitro* histone methyltransferase assay was performed using recombinant proteins. The full-length coding sequences of BcSET2 and *BcEaf2* were cloned into expression vectors and heterologously expressed in *Escherichia coli*. Recombinant proteins were purified by affinity chromatography using Ni-NTA agarose (Qiagen) and verified by SDS-PAGE. Methyltransferase reactions were carried out in a 30 μL system containing purified BcSET2 (0.5–1 μg), with or without BcEaf2 (equimolar ratio), recombinant histone H3 protein or nucleosome substrates (1–2 μg), and 50 μM S-adenosyl-L-methionine (SAM) as the methyl donor, in methyltransferase buffer (50 mM Tris-HCl, pH 8.0, 5 mM MgCl_2_, 1 mM DTT). Reactions were incubated at 30 °C for 1–2 h and terminated by the addition of SDS loading buffer followed by boiling for 5 min. Reaction products were resolved by SDS-PAGE and analyzed by immunoblotting using anti-H3K36me3-specific antibodies to detect methylation levels. Total histone H3 levels were detected using anti-H3 antibodies as loading controls. Band intensities were quantified using ImageJ software to compare relative methyltransferase activity. All assays were performed with at least three independent biological replicates.

### 4.11 Phylogenetic analysis

Phylogenetic analysis was performed using MEGA 7 software. Protein sequences of the selected homologous proteins were aligned using the ClustalW algorithm implemented in MEGA 7. The phylogenetic tree was constructed using the neighbor-joining (NJ) method with 1,000 bootstrap replicates to assess the reliability of the inferred phylogenetic relationships. The generated phylogenetic tree was visualized and edited using MEGA 7.

### 4.12 Statistical analysis

All experiments were performed with at least three independent biological replicates, and data are presented as the mean ± standard deviation (SD). Statistical analyses were performed using GraphPad Prism (if applicable) or the indicated statistical methods described in the figure legends. Differences among multiple groups were analyzed using one-way analysis of variance (one-way ANOVA), with different letters indicating statistically significant differences. Comparisons between two groups were performed using Student’s *t*-test or unpaired two-tailed *t*-test. ImageJ software was used for densitometric analysis of immunoblot signals. MEGA 7 software was used for phylogenetic tree construction. A value of *P* < 0.05 was considered statistically significant.

## Data Availability

The datasets presented in this study can be found in online repositories. The names of the repository/repositories and accession number(s) can be found in the article/Supplementary material.

## Footnotes

1
http://www.broadinstitute.org/
