# Supplementary material for: BcEaf2 plays a critical role in hyphal growth, asexual reproduction, pathogenic infection, and histone methylation in Botrytis cinerea
Source: Front Microbiol. 2026 Oct 20;17:1978891. doi: 10.3389/fmicb.2026.1978891 (PMC13727723; doi:10.3389/fmicb.2026.1978891)
Supplement: Supplementary file 1 [file Data_Sheet_1.docx]

**Supplementary Materials For**

**Novel function of the in regulating the growth, asexual reproduction, pathogenicity, and histone methylation of *Botrytis cinerea*^1^**

WU Junwei^1^,WU Weigang^1^,WU Yajing^2^,HAO Jianyu^3^,CHEN Wenchao^3^,WANG Haixu^1^,YANG Chen^1^,SHEN Fengying^1*^

1. Hebei North University, Zhangjiakou 075000, China 2.Hebei Academy of Forestry and Grassland Science,Shijiazhuang 050061,China;3.Zhangjiakou Academy of Agricultural Sciencesy, Zhangjiakou 075000, China

***Authors for correspondence:** SHEN Fengying	E-mail:zhibao200221@163.com

**The file includes:**

Figs. S1 to S5

Legends for Fig. S1 to S5

**Fig. S1 Generation of *BcEaf2* silencing mutants.**

**(A)** Schematic diagram of gene silencing. For *BcEaf2* silencing, a reverse-complementary sequence targeting the SANT domain-coding region of BcEaf2 was cloned into the gene-silencing vector to generate the *BcEaf2*-silenced strains (siBcEaf2-1, siBcEaf2-2, and siBcEaf2-3). The corresponding complemented strain was designated siBcEaf2-C. **(B)** Relative expression of *BcEaf2* gene in silencing mutants and complemented strain siBcEaf2-C compared to WT. The asterisks indicate significant differences based on Tukey′s test (**P < 0.01). The error bars indicate standard deviations (n = 3).

**Fig. S2 *BcEaf2* silencing affects stress tolerance and conidial production in *B. cinerea***

**(A**) Mycelial growth of the WT (B05.10), empty vector (EV), *BsEaf2*-silenced (siBcEaf2-1, siBcEaf2-2, and siBcEaf2-3), and siBcEaf2-C strains incubated for 3 days under different growth conditions. **(B)** Mycelial inhibition by the 0.5 M KCl or 0.5 M Sorbitol relative to untreated control was calculated.  (**C**) Conidial production of the indicated strains was quantified after 7 and 11 days of incubation on PDA plates. Conidia were harvested from fungal colonies, and the number of conidia was determined using a hemocytometer. The conidial yields of the wild-type (WT), *BcEaf2*-silenced strains, and complemented strain were compared at the indicated time points. Data in A-C are presented as the mean ± standard deviation from three biological replicates. Statistical significance was determined according to Student’s t-test (**P < 0.01).

**Fig. S3 Homologous recombination-mediated gene knockout of *BcSET2* genes.**

**(A)** Relative expression of *BcSET2* gene in *siBcEaf2* silencing mutants and complemented strain siBcEaf2-C compared to WT. **(B)** Western blot analysis of BcSET2 protein levels in WT and BcSET2^Mut^. Tubulin was used as the loading control. **(C)** Quantification of BcSET2 protein abundance based on the band intensities measured using ImageJ. **(D)** Analysis of genomic DNA from the WT (B05.10), empty vector (EV), and *BcSET2* knockout mutants (Δ*BcSET2*-1, Δ*BcSET2*-2 and Δ*BcSET2*-3) and the corresponding complemented (Δ*BcSET2*-C) using the primers shown in (A). F1/R1 was used to verify whether homologous substitution has occurred, while F2/R2 was used to confirm whether the target gene has been successfully knocked out. **(E)** Relative expression of *BcSET2* in above strains compared to WT. The asterisks indicate significant differences based on Tukey′s test (**P < 0.01). The error bars indicate standard deviations (n = 3).The asterisks indicate significant differences based on Tukey′s test (**P < 0.01). Data are presented as means ± SD from three independent biological replicates.

**Fig. S4 BcSET2 is required for mycelial growth, stress tolerance, and conidial production in *B. cinerea***

(A) Colony diameters of WT, GFP-BcEaf2, and FLAG-BcSET2 strains were measured at the indicated time points, and colony growth was quantified. Data are presented as mean ± SD from three independent biological replicates. (B) Virulence of WT, GFP-BcEaf2, and FLAG-BcSET2 strains was evaluated on detached leaves of *B. cinerea* host plants. Representative lesions were photographed at the indicated time point, and lesion areas were quantified. Data are presented as mean ± SD from three independent biological replicates. Statistical significance was determined by one-way ANOVA with Tukey’s multiple-comparison test. Different letters indicate significant differences (P < 0.05). Mycelial growth of WT (B05.10), empty vector (EV), and *BcSET2* knockout mutants (Δ*BcSET2*-1, Δ*BcSET2*-2 and Δ*BcSET2*-3) and the corresponding complemented (Δ*BcSET2*-C) incubated for 3 days under different growth conditions. Mycelial inhibition by the 10 mM H_2_O_2_ **(C)**, 0.5 M KCl **(D)** or 0.5 M Sorbitol **(E)** relative to untreated control was calculated. (**F**) Conidial production of the indicated strains was quantified after 7 and 11 days of incubation on PDA plates. Conidia were harvested from fungal colonies, and the number of conidia was determined using a hemocytometer. The conidial yields of the wild-type (WT), EV, *BcSET2* knockout strains, and complemented strain were compared at the indicated time points. Data in A-D are presented as the mean ± standard deviation from three biological replicates. Statistical significance was determined according to Student’s t-test (**P < 0.01).

**Fig. S5 *BcEaf2-*silenced and *BcSET2* knockout mutants exhibited increased sensitivity to DNA damaging agents MMC.**

(A) *BcEaf2-*silenced and *BcSET2* knockout mutants exhibited increased sensitivity to DNA damaging agents MMC. Mycelial growth of WT (B05.10), empty vector (EV), *BcEaf2-*silenced mutants (siBcEaf2-1, siBcEaf2-2, and siBcEaf2-3), the corresponding complemented (siBcEaf2-C), BcSET2 knockout mutants (Δ*BcSET2*-1, Δ*BcSET2*-2 and Δ*BcSET2*-3) and the corresponding complemented (Δ*BcSET2*-C) on PDA agar medium in the presence of 10 ppm MMC. Mycelial growth of above strains on PDA agar medium with 0.1% DMSO was used as control. (B) Inhibition ratio was calculated as (Control−Growth rate on plates with treatment)/Control growth×100% after 3 days. Statistical significance compared to the wild-type was determined using Student's t-test (**P < 0.01, ns: not significant). Data in (B) are presented as the mean ± standard deviation from three biological replicates.
